# The cAMP Response Element- Binding Protein/Brain-Derived Neurotrophic Factor Pathway in Anterior Cingulate Cortex Regulates Neuropathic Pain and Anxiodepression Like Behaviors in Rats

**DOI:** 10.3389/fnmol.2022.831151

**Published:** 2022-03-24

**Authors:** Jing Wen, Yaowei Xu, Zhixiang Yu, Yifan Zhou, Wenting Wang, Jingjie Yang, Yiming Wang, Qian Bai, Zhisong Li

**Affiliations:** ^1^Department of Anesthesiology and Perioperative Medicine, Second Affiliated Hospital of Zhengzhou University, Zhengzhou, China; ^2^Institute of Neuroscience, Academy of Medical Sciences, Zhengzhou University, Zhengzhou, China

**Keywords:** neuropathic pain, anxiety, depression, CREB, BDNF, anterior cingulate cortex

## Abstract

Neuropathic pain is often accompanied by anxiety and depression-like manifestations. Many studies have shown that alterations in synaptic plasticity in the anterior cingulate cortex (ACC) play a critical role, but the specific underlying mechanisms remain unclear. Previously, we showed that cAMP response element-binding protein (CREB) in the dorsal root ganglion (DRG) acts as a transcription factor contributing to neuropathic pain development. At the same time, brain-derived neurotrophic factor (BDNF), as important targets of CREB, is intricate in neuronal growth, differentiation, as well as the establishment of synaptic plasticity. Here, we found that peripheral nerve injury activated the spinal cord and ACC, and silencing the ACC resulted in significant relief of pain sensitivity, anxiety, and depression in SNI rats. In parallel, the CREB/BDNF pathway was activated in the spinal cord and ACC. Central specific knockdown and peripheral non-specific inhibition of CREB reversed pain sensitivity and anxiodepression induced by peripheral nerve injury. Consequently, we identified cingulate CREB/BDNF as an assuring therapeutic method for treating neuropathic pain as well as related anxiodepression.

## Introduction

Neuropathic pain results from damage or disease to the somatosensory nervous system, characterized by allodynia, hyperalgesia, and spontaneous pain (Xu et al., 2021). At the same time, the transmission of long-term nociceptive signals induces plasticity changes in the structure and function of some brain regions such as the medial prefrontal cortex (mPFC) (Mecca et al., 2021), ACC (Smith et al., 2021), amygdala (Murugappan et al., 2021), and hippocampus (Toledo et al., 2021), which are also involved in the regulation of emotion and cognitive functions, so that nociceptive signals not only include pain components but also cover emotion-related components in the brain (De Ridder et al., 2021). Clinical studies also point out that patients with neuropathic pain are often comorbid with disorders of emotional function (Fonseca-Rodrigues et al., 2021), and patients with anxiety and depression often have allodynia and hyperalgesia (Corlier et al., 2021), which exacerbates the vicious circle between pain and psychiatric disorders. Therefore, developing a novel and effective therapy for mental illnesses associated with neuropathic pain remains a significant issue.

The anterior cingulate cortex (ACC) is an information processing region in the limbic system that receives nociceptive information projecting from the thalamus and somatosensory cortex (Marti Mengual et al., 2020; Bliss-Moreau et al., 2021; Wang Y. et al., 2021), as well as fear/anxiety information projecting from the amygdala (Shao et al., 2021). This unique property enables ACC neurons to integrate sensory inputs and emotional signals, which plays an important role in pain and interdependent anxiodepression-like behaviors. Clinical MRI has shown decreased gray matter volume, and increased hemodynamic signal in the ACC in patients with neuropathic pain and emotional dysfunction (Da Silva et al., 2020; McIlwrath et al., 2020), and disruption of abnormal ACC signal by bilateral anterior cingulate transection (Deng et al., 2019), deep brain stimulation (DBS) (Boccard et al., 2014), or transcranial direct current stimulation can treat neuropathic pain and major depressive disorder (Riva-Posse et al., 2014). Local silencing or restraining long-term potentiation (LTP) in the ACC effectively reduces neuropathic pain as well as anxiodepression-like behaviors (Li et al., 2021). It also has been shown that the ACC can project directly to the dorsal horn of the spinal cord and exerts facilitatory effects on the transmission of pain signals (Chen et al., 2018). Although the ACC has been significantly associated with pain and mental illnesses, the molecular mechanisms by which nociceptive transmission activates it and results in aberrant synaptic plasticity remain unknown.

Peripheral nerve injury inflicts nociceptive signals that are constantly transmitted across synapses. As a member of transcription factors (Saura and Cardinaux, 2017), cAMP-response element-binding protein (CREB), mediates inter-synaptic nociceptive signaling into the cell by effectively regulating gene transcription, which induces the immediate to long-lasting conversion in nociceptive signaling (Carrano et al., 2019). A considerable increase in the transcriptional activity of p-CREB, which migrates into the nucleus and stimulates the transcription of target genes with a cyclic adenosine monophosphate response element (CRE) motif, such as brain-derived neurotrophic factor (BDNF) (Navabpour et al., 2021). BDNF is critical for nervous system activity and function, and plays a part in the formation of pain (Huang L. et al., 2021), memory (Sun W. et al., 2021), cognition (Tomoda et al., 2021), and mood (Ge et al., 2019).

BDNF is up-regulated in the ACC of rats with inflammatory pain, and injection of recombinant BDNF or adeno-associated viral vectors into the ACC triggers neuronal hyperexcitability and induces persistent pain hypersensitivity (Thibault et al., 2014; Huang L. et al., 2021). Meanwhile, peripheral nerve injury-induced the generation of silent synapses and increased synaptic plasticity by promoting the expression of BDNF in the ACC (Wang I. et al., 2021). The CREB/BDNF pathway plays an important role in both pain and depression. However, little is known about whether and how the CREB/BDNF pathway in the spinal cord and ACC involves neuropathic pain and related anxiodepression.

In our study, Combination of behavioral, biochemical, viral tracing and gene silencing methods were used to uncover a critical part of CREB/BDNF in the ACC in neuropathic pain as well as related anxiodepression. We proved that peripheral nerve injury results in depression-like behaviors as well as anxiety. Meanwhile, the spinal cord and ACC are abnormally activated in the SNI model. Furthermore, CREB/BDNF expression are increased in the spinal cord and ACC in SNI rats. Silencing ACC by chemical genetic approaches and knocking down the expression of CREB/BDNF in the ACC both alleviate pain sensitivity and anxiodepression like behaviors. At the same time, we inhibited neuropathic pain progress in the initial phase, which also alleviated anxiodepression-like behaviors. Here, we identify CREB/BDNF as one of the critical molecules that participate in the development of neuropathic pain and depression, and found that the mechanism of central sensitization resembles the ACC and spinal.

## Results

### Peripheral Neuropathy Induces Anxiety and Depression Like Behaviors in Rats

**FIGURE 1:**
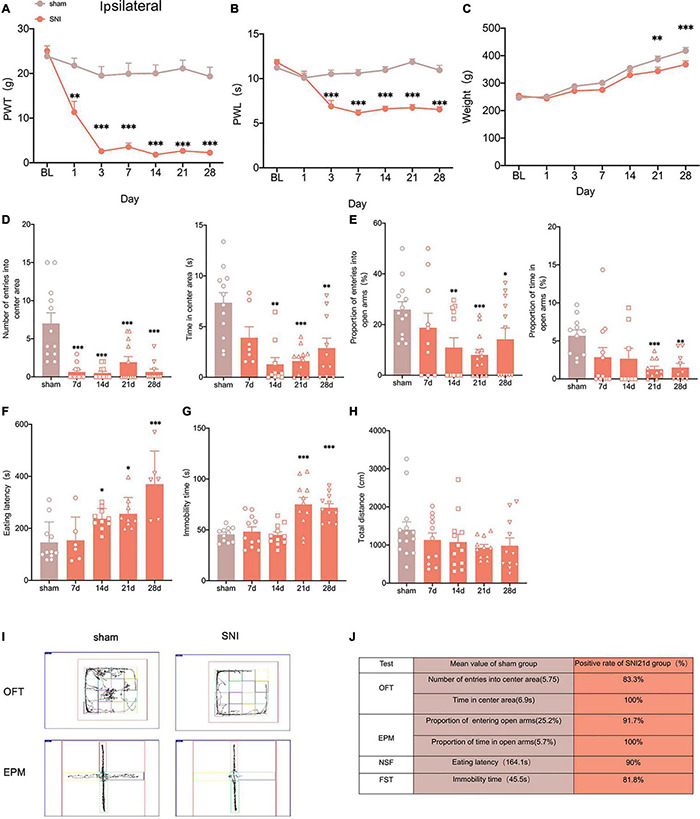
Selectively nerve injured rats exhibit pain hypersensitivity and anxiodepression like behaviors. **(A,B)** SNI significantly reduced the PWT **(A)** and the PWL **(B)** in the ipsilateral paws (*n* = 8 rats/group). **(C)** The weight growth rate of SNI rats was lower than that of sham rats (*n* = 8 rats/group). **(D)** The rats showed a significant reduction in the number of entries into the center area and the time spent in the center area after SNI in the OFT (*n* = 12 rats/group). **(E)** The proportion of entries into the open arms and the proportion of time in the open arms were significantly decreased in the SNI rats in the EPM (*n* = 12 rats/group). **(F,G)** The eating latency in the NFST **(F)** and the immobility time in the FST **(G)** of SNI rats were significant increase of SNI rats (*n* = 12 rats/group). **(H)** There was no significant difference in the total distance traveled by SNI rats in the OFT (*n* = 12 rats/group). **(I)** Schematic representation of the locomotor trajectories of sham and SNI rats at 21 days after surgery in the OFT and EPM. **(J)** Probability of SNI rats exhibiting anxiety and depression like behaviors at 21 days after surgery. *P < 0.05, ^**^P < 0.001, ^***^P < 0.0001 for sham vs. SNI.

To construct neuropathic pain, we used the selective nerve injury (SNI) model (Zhao et al., 2017). The SNI produced robust mechanical allodynia and thermal hyperalgesia at 7 days, stabilizing posteriorly (Figures 1A,B). However, there was no significant change in the contralateral paws (Supplementary Figure 1A). We also found that the weight of rats in the SNI group was significantly less than that in the sham group at 21 and 28 days after surgery (Figure 1C), which is in line with the results of clinical studies that weight loss in patients with anxiety and depression (Huang H. et al., 2021).

To explore whether the SNI model would exhibit anxiety and depression like behaviors, we conducted several anxiodepressive-like behaviors tests. In the open field test (OFT), SNI rats showed a significant decrease in the number of entries into the central area and time in the central area at 14 days, compared with the sham group (Figure 1D). At the same time, in the elevated plus maze test (EPM), SNI rats also showed a significant reduction in the proportion of entries into the open arms at 14 days (Figure 1E). The novelty suppressed feeding test (NSFT) measures depression and anxiety by forcing rats to choose between eating and escaping in a novel environment. At 14 days after SNI, rats showed a significant increase in eating latency than the sham group (Figure 1F). However, our results showed that the immobility time of rats significantly increased at 21 days but not 14 days after SNI in the forced swimming test (FST) (Figure 1G). In addition, the locomotor activity of the SNI rats was not affected (Figure 1H). In summary, the SNI model showed anxiety-like behavior as early as 14 days and depression-like behavior as early as 21 days after surgery (Figure 1I).

Clinical studies indicate that not all patients with neuropathic pain share comorbidity (Kec et al., 2021), so we assessed the probability of comorbid anxiety and depression in SNI rats by comparing with the sham group in each experimental index and found that at least 80% of the rats developed anxiety and depression- like behaviors at 21 days after SNI (Figure 1J). So, peripheral nerve injury can induce anxiety and depression in rats with high probability.

### Anterior Cingulate Cortex Activation Induces Pain Hypersensitivity and Anxiodepression in Selective Nerve Injury Rats

**FIGURE 2:**
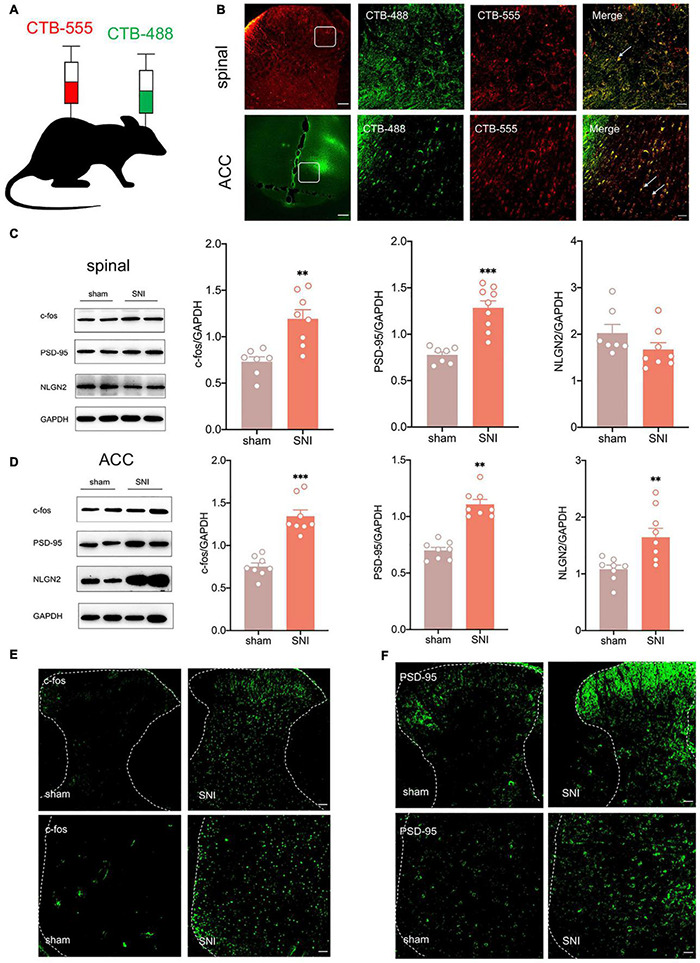
There is a reciprocal projection between the ACC and the spinal cord, and peripheral nerve injury activates the spinal cord and the ACC. **(A)** CTB-555 (green, 250 nl) and CTB-488 (red, 250 nl), respectively, were stereotaxically injected into the ipsilateral spinal dorsal horn and contralateral ACC (Scar bar = 50 μm). **(B)** Immunofluorescence showed that coexpression signals (yellow) of CTB-555 and CTB-488 were observed in the injected area of the spinal dorsal horn and ACC. **(C)** The expression of c-fos and PSD-95 was increased in the spinal cord of SNI rats (*n* = 8 rats/group). **(D)** The expression of c-fos, PSD-95 and NLGN2 was increased in the ACC of SNI rats (*n* = 8 rats/group). **(E)** Peripheral nerve injury activated ipsilateral spinal cord and contralateral ACC (Scar bar = 50 μm). **(F)** Peripheral nerve injury elevated PSD-95 in the ipsilateral spinal cord and contralateral ACC (Scar bar = 50 μm). ^**^P < 0.001, ^***^P < 0.0001 for sham vs. SNI.

According to Chen et al. (2018), the ACC may facilitate the top-down transmission of pain, and there is a direct projection relationship between the ACC and the spinal cord. To demonstrate the role of the ACC in neuropathic pain and comorbidity, we first wanted to verify the anatomical connection between the ACC and the spinal cord. Cholera toxin subunit B (CTB), taken up by axon terminals and retrogradely transported to the soma, is currently the most widely used retrograde tracer (Yang M. et al., 2021). We microinjected CTB-555(250 nl, green) into the contralateral ACC and CTB-488(250 nl, red) into the ipsilateral spinal dorsal horn (Figure 2A). We observed that both CTB-555 and CTB-488 appeared to be coexpressed in the ACC and spinal dorsal horn after 3 days, suggesting a direct reciprocal projection between the ACC and spinal cord anatomically (Figure 2B).

Next, we wanted to explore whether ACC was activated in SNI rats. As an immediate expression gene, c-fos is often used as a marker of neuronal activity (Johnson et al., 2021). Western blotting showed that c-fos and postsynaptic density-95 (PSD-95) expression were increased in the ipsilateral spinal cord and contralateral ACC, and neuroligin 2 (NLGN2) expression was increased in the ACC at 21 days after SNI (Figures 2C,D), suggesting that the spinal cord and ACC were activated and synapse construction were increased after peripheral nerve injury. Immunofluorescence also demonstrated elevation of c-fos and PSD-95 (Figures 2E,F). Taken together, pain signals may be transmitted from the dorsal horn of the spinal cord to the ACC by spinal-ACC projections, activate the ACC, and induce synaptic remodeling in the spinal cord and ACC.

**FIGURE 3:**
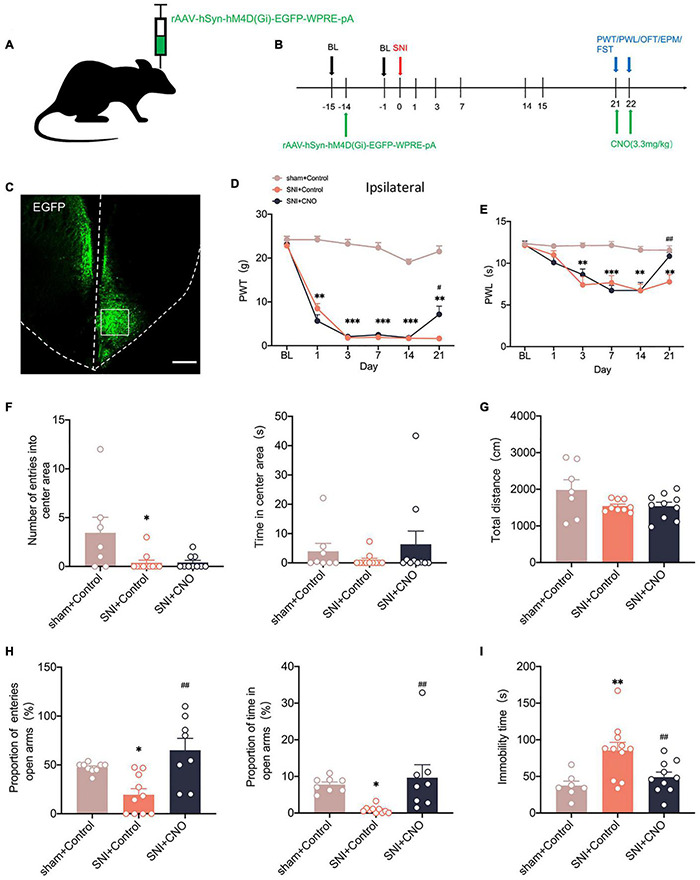
Silencing the ACC by chemicalgenetic techniques reduced neuropathic pain and anxiodepression like behaviors. **(A,B)** Injection of rAAV-hSyn-hM4D (Gi)-EGFP into the contralateral ACC of SNI rats **(A)** and experimental timing **(B)**. **(C)** Immunofluorescence showed that rAAV-hSyn-hM4D (Gi)-EGFP was injected into the ACC and was successfully transfected (Scar bar = 50 μm). **(D,E)** Intraperitoneal injection of CNO (3.3 mg/kg) to silence ACC attenuated PWT **(D)** and PWL **(E)** in SNI rats (*n* = 10 rats/group). **(F,G)** Intraperitoneal injection of CNO (3.3 mg/kg) to silence ACC did not influence the number of entries into the center area, the time in the center area **(F)**, or the total distance **(G)** in the OFT (*n* = 10 rats/group). **(H)** Silencing ACC by intraperitoneal injection of CNO (3.3 mg/kg) increased the proportion of entries into the open arms and the proportion of time in the open arms in the EPM (*n* = 10 rats/group). **(I)** Intraperitoneal injection of CNO (3.3 mg/kg) to silence ACC decreased the immobility time in the FST (*n* = 10 rats/group). *P < 0.05, ^**^P < 0.001, ^***^P < 0.0001 for sham + Control vs. SNI + Control; *^#^P* < 0.05, *^##^P* < 0.001 for SNI + Control vs. SNI + CNO.

We used chemicalgenetic techniques to non-specifically silence the ACC to investigate its contribution to neuropathic pain and emotional dysfunction after SNI. A chemicalgenetic virus (rAAV-hM4Di-EGFP) was injected into the right ACC preoperatively, and clozapine N-oxide (CNO, 3.3 mg/kg) was administered intraperitoneally to specifically activate the virus to silence ACC, followed by relevant behavioral tests (Figures 3A,B). At 14 days after SNI, immunofluorescence showed that the virus was injected in the contralateral ACC and stably transfected (Figure 3C). Mechanical paw withdrawal threshold (PWT) and thermal paw withdrawal latency (PWL) were significantly reduced in SNI rats (Figures 3D,E). Both PWT and PWL significantly increased after CNO intraperitoneal injection (Figures 3D,E). However, there was no significant change in the contralateral paws (Supplementary Figure 1B). In parallel, anxiety and depression like behaviors were observed in SNI rats (Figures 3F–I). At the same time, rats in the SNI + CNO group showed a significant increase in the proportion of entries into the open arms and the proportion of time in the open arms compared with the SNI + control group in the EPM (Figure 3H). Silencing ACC also significantly reduced the immobility time of SNI rats in the FST (Figure 3I). However, no effect was observed in the OFT. Meanwhile, the locomotor activity of rats in each group was not affected (Figures 3F,G). Therefore, silencing ACC reduced the pain hypersensitivity and anxiodepression in SNI rats to some extent.

### Peripheral Nerve Injury Activates the cAMP Response Element-Binding Protein/Brain-Derived Neurotrophic Factor Pathway in the Spinal Cord and Anterior Cingulate Cortex

**FIGURE 4:**
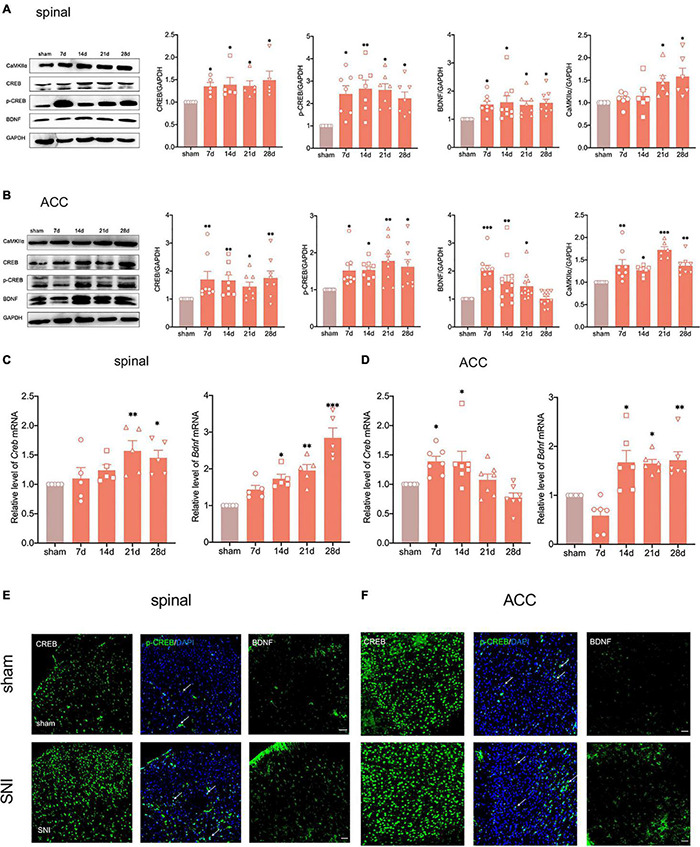
Peripheral nerve injury activates the CREB/BDNF pathway in the spinal cord and ACC. **(A)** The expression of CREB, p-CREB, BDNF and CaMKII α was significantly increased in the ipsilateral spinal cord at all-time points (7, 14, 21, and 28 days) after the nerve injury in rats (*n* = 8 rats/group). **(B)** The expression of CREB, p-CREB, BDNF and CaMKII α was significantly increased in the contralateral ACC at all-time points (7, 14, 21, and 28 days) after the nerve injury in rats (*n* = 8 rats/group). **(C)** The expression of CREB mRNA and BDNF mRNA was significantly increased in the spinal cords of SNI rats (*n* = 5 rats/group). **(D)** The expression of CREB mRNA and BDNF mRNA was significantly increased in the ACC of SNI rats (*n* = 7 rats/group). **(E,F)** Immunofluorescence showed that peripheral nerve injury induced an increase in the expression of CREB, p-CREB, and BDNF in the ipsilateral spinal cord **(E)** and contralateral ACC **(F)** (Scar bar = 50 μm). *P < 0.05, ^**^P < 0.001, ^***^P < 0.0001 for sham vs. SNI.

We have shown that elevated CREB in dorsal root ganglion (DRG) promotes the chemotherapy-induced pain in a previous study (Yang Y. et al., 2021). Concurrently, BDNF, as one of the downstream targets of CREB, plays a vital role in neurological diseases (Liao et al., 2020). To explore whether the CREB/BDNF signaling pathway plays a role in neuropathic pain and anxiodepression, we first used western blotting to examine the expression of CREB/BDNF in the spinal cord and ACC after peripheral nerve injury. The results showed that the expression of CREB, p-CREB, BDNF and calcium/calmodulin dependent protein kinase II α (CaMKIIα) in the ipsilateral spinal cord was significantly increased in SNI rats (Figure 4A). At the same time, the phosphorylation of CREB in spinal cord of SNI group increased (Supplementary Figure 3C). In addition, it showed that BDNF mRNA began to increase as early as 7 days, whereas the CREB mRNA increased at 14 days after surgery (Figure 4C).

The expression of CREB, p-CREB, and CaMKIIα in the contralateral ACC was similar to that in the spinal cord (Figure 4B and Supplementary Figure 3C). Meanwhile, the CREB mRNA increased in the early postoperative period at 7 and 14 days (Figure 4D). Interestingly, the expression of BDNF increased up to 21 days and returned to normal levels at 28 days (Figure 4B). However, BDNF mRNA still significantly increased at 28 days after SNI (Figure 4D). To investigate this phenomenon, we selected the contralateral hippocampus of SNI rats. The results showed that the CREB/BDNF pathway was not significantly affected in the hippocampus of SNI rats without depression. However, it was suppressed in comorbid rats (Supplementary Figure 1E). We therefore speculated that locally increased BDNF in the ACC may be secreted to other brain regions (such as the hippocampus) as a compensatory mechanism after depression-like behaviors appear.

**FIGURE 5:**
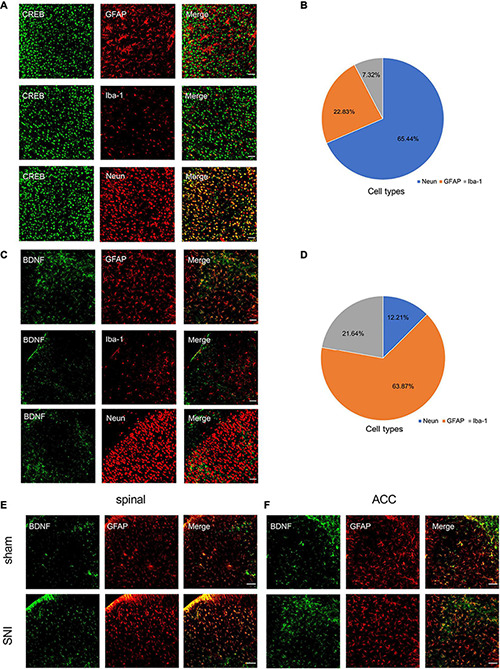
Characterization of expression profile of CREB and BDNF in the ACC. **(A,B)** Representative examples **(A)** and quantitative summary **(B)** showing that CREB was highly coexpressed with neuronal nuclear antigen (NeuN), sparsely with either glial fibrillary acidic protein (GFAP) or Iba1 (*n* = 3) (Scar bar = 50 μm). **(C,D)** Representative examples **(C)** and quantitative summary **(D)** showing that BDNF was highly coexpressed with glial fibrillary acidic protein (GFAP), sparsely with either neuronal nuclear antigen (NeuN) or Iba1 (*n* = 3) (Scar bar = 50 μm). **(E,F)** Peripheral nerve injury increases the coexpression of BDNF and GFAP in the spinal cord **(E)** and ACC **(F)** (Scar bar = 50 μm).

Immunofluorescence staining showed that SNI significantly increased CREB, p-CREB, and BDNF expression in the spinal cord and ACC at 21 days after SNI (Figures 4E,F). We also found that p-CREB has two different expression morphologies: cytoplasmic and nuclear, and SNI increased the expression of both forms (Figures 4E,F). We then characterized the expression profile of CREB and BDNF in the ACC. Double immunofluorescence staining revealed that CREB immunoreactivity was highly coexpressed with neuronal nuclear antigen (NeuN), sparsely with either glial fibrillary acidic protein (GFAP) or Iba1 (Figures 5A,B). However, BDNF immunoreactivity was highly coexpressed with GFAP in the ACC, but not NeuN, and Iba1 (Figures 5C,D), which suggested that the source of BDNF in ACC may be mainly in astrocytes, and SNI also increased the labeling signal between BDNF and GFAP in the spinal cord and ACC (Figures 5E,F). At the same time, we used double immunofluorescence to evaluate the co-labeling of CREB and BDNF in ACC, and found that there were few co-labeling signals between CREB and BDNF (Supplementary Figure 3M). BDNF, as a secretory protein, mostly exists outside the cell, so it is less co-labeled with CREB in the nucleus. Therefore, it may be more accurate to use Chromatin Immunoprecipitation to evaluate the binding between the two. In conclusion, SNI increased CREB/BDNF expression in the ipsilateral spinal cord and contralateral ACC, and promoted central sensitization, allowing more pain signals to be transmitted to the brain, resulting in structural and functional disturbances in various brain regions.

### Knockdown of cAMP Response Element-Binding Protein in the Anterior Cingulate Cortex Alleviates Neuropathic Pain and Anxiodepression

**FIGURE 6:**
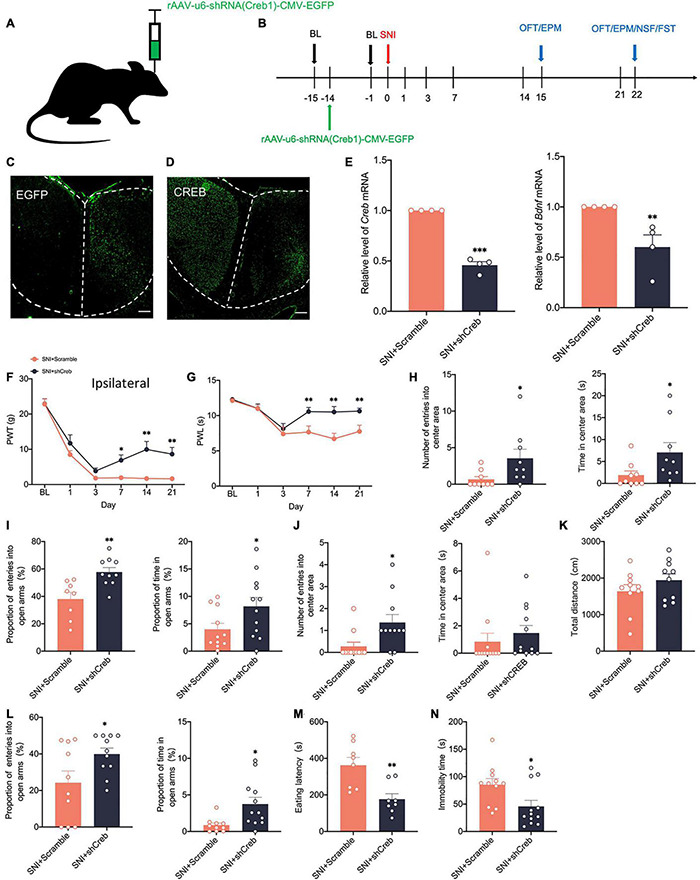
Knockdown of CREB in the ACC alleviates pain hypersensitivity and anxiodepression like behaviors in SNI rats. **(A,B)** Injection of rAAV-u6-shRNA Creb1-CMV-EGFP into the contralateral ACC of SNI rats **(A)** and experimental timing **(B)**. **(C)** The virus was successfully injected into the ACC and stably transfected (Scar bar = 50 μm). **(D)** CREB was significantly knocked down within the ACC (Scar bar = 50 μm). **(E)** Knockdown of CREB in the ACC decreased CREB/BDNF mRNA in the ACC (*n* = 4 rats/group). **(F,G)** Knockdown of CREB in the ACC alleviated PWT **(F)** and PWL **(G)** in SNI rats (*n* = 12 rats/group). **(H)** Knockdown of CREB in the ACC increased the number of entries into the center area and time in the center area in SNI rats at 14 days after surgery (*n* = 12 rats/group). **(I)** Knockdown of CREB in the ACC increased the proportion of entries into the open arms and the proportion of time in the open arms in SNI rats at 14 days after surgery (*n* = 12 rats/group). **(J)** Knockdown of CREB in the ACC increased the number of entries into the center area, but not the time in the center area in SNI rats at 21 days after surgery (*n* = 12 rats/group). **(K)** Knockdown of CREB in the ACC was no significant difference in the total distance traveled by SNI rats at 21 days after surgery (*n* = 12 rats/group). **(L)** Knockdown of CREB in the ACC increased the proportion of entries into the open arms and the proportion of time in the open arms in SNI rats at 21 days after surgery (*n* = 12 rats/group). **(M,N)** Knockdown of CREB in the ACC decreased the eating latency **(M)** and the immobility time **(N)** in SNI rats at 21 days after surgery (*n* = 12 rats/group). *P < 0.05,^**^P < 0.001,^***^P < 0.0001 for SNI + Scramble vs. SNI + shCreb.

To address whether there is a causal relationship of activity-dependent changes of CREB/BDNF with neuropathic pain and anxiodepressive consequences, we generated recombinant adeno-associated virus (AAV2/9) expressing both a small interfering hairpin RNA (shRNA) targeted against CREB (AAV-shCreb) and EGFP. To ensure the efficiency of virus transfection, we performed stereotaxic injections at 14 days before SNI (Figures 6A,B). At 21 days after viral injection, immunofluorescence showed that CREB expression was significantly reduced when the virus was stably transfected in the contralateral ACC (Figures 6C,D). We also found that the mRNA of CREB and BDNF was significantly decreased in the contralateral ACC (Figure 6E). The knockdown of CREB in the ACC significantly reduced PWT and PWL in SNI rats (Figures 6F,G). However, there was no significant change in the contralateral paws (Supplementary Figure 1C). Meanwhile, the knockdown of CREB in the ACC reversed the anxiety like behaviors induced by SNI in the EPM OFT and EPM at 14 days (Figures 6H,I). At 21 days after SNI, the knockdown of CREB in the ACC increased the proportion of entries into the open arms and time in the open arms (Figure 6L). The eating latency and immobility time were also significantly decreased in the SNI + shCreb group (Figures 6M,N). Surprisingly, the open field test was not affected, and we speculated that it might be due to the behavioral tests of 14 days that decreased the interest of rats in novel environment exploration (Figure 6J). The locomotion capacity of rats in each group was not changed (Figure 6K). In summary, knockdown of CREB in the ACC alleviates anxiety and depression behaviors induced by peripheral nerve injury. However, after knocking down CREB in the ACC of sham group, rats did not show prominent pain hypersensitivity and anxiodepression like behavior (Supplementary Figures 2A–J).

**FIGURE 7:**
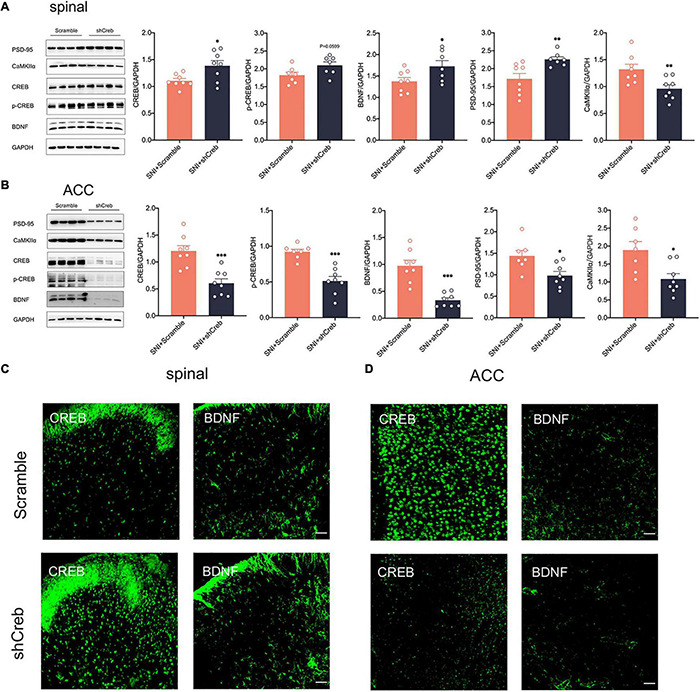
Knockdown of CREB in the ACC increased CREB/BDNF expression in the spinal cord in SNI rats. **(A)** Knockdown of CREB in the ACC activated the CREB/BDNF pathway in the spinal cord (*n* = 8 rats/group). **(B)** Knockdown of CREB in the ACC inhibited the CREB/BDNF pathway in the ACC (*n* = 8 rats/group). **(C)** Representative examples showing that knockdown of CREB in the ACC increased the expression of CREB and BDNF in the spinal cord (Scar bar = 50 μm). **(D)** Representative examples showing that knockdown of CREB in the ACC decreased the expression of CREB and BDNF in the ACC (Scar bar = 50 μm). *P < 0.05, ^**^P < 0.001, ^***^P < 0.0001 for SNI + Scramble vs. SNI + shCreb.

To explore whether knockdown of CREB within the ACC has an effect on the CREB/BDNF pathway in the spinal cord, we took Real-time quantitative PCR, western blotting, and immunofluorescence to explore these changes. It is intriguing that knockdown of CREB in the ACC, however, increased the expression of CREB, p-CREB, BDNF and PSD-95 in the spinal cord (Figures 7A,C), possibly suggesting that BDNF exerted its neurotrophic effects to promote injury peripheral nerve repair. To explore the impact of blocking the middle ring of pain signal neural circuit on the upstream and downstream is our next focus. However, the mRNA of CREB and BDNF was not affected in the spinal cord of SNI + shCreb rats (Supplementary Figure 1F). Also, CREB, p-CREB, BDNF, PSD-95 and CaMKIIα were significantly reduced in the ACC of SNI + shCreb rats (Figures 7B,D), but it did not affect the phosphorylation degree of CREB in the spinal cord and ACC (Supplementary Figure 3D), suggesting that the CREB/BDNF pathway was inhibited and excitatory synapses were reduced. In conclusion, inhibition of the CREB/BDNF pathway in the ACC reduces pain signaling in the ACC and, in turn, alleviates neuropathic pain and anxiodepression in SNI rats.

### Intraperitoneal cAMP Response Element-Binding Protein Inhibitor Reduces Pain Hypersensitivity and Anxiodepression in Selective Nerve Injury Rats

**FIGURE 8:**
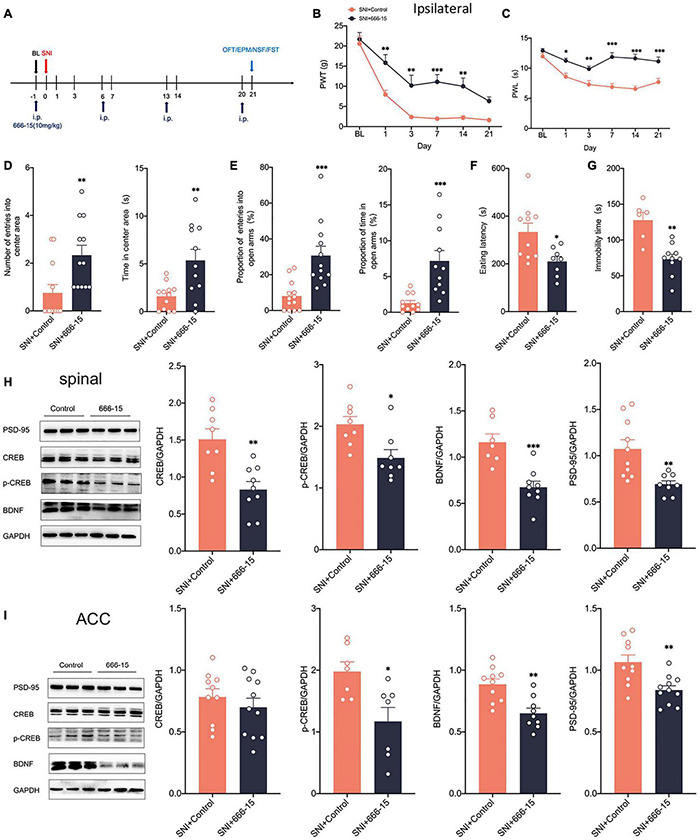
Intraperitoneal administration of the CREB inhibitor (666–15) reduced neuropathic pain and anxiodepression like behaviors in SNI rats. **(A)** 666–15 (10 mg/kg) was administered intraperitoneally 1 day before surgery and at 7 days intervals thereafter. **(B,C)** Intraperitoneal injection of 666–15 attenuated PWT **(B)** and PWL **(C)** in SNI rats (*n* = 12 rats/group). **(D)** Intraperitoneal injection of 666–15 increased the number of entries into the center area and the time in the center area in SNI rats (*n* = 12 rats/group). **(E)** Intraperitoneal injection of 666–15 increased the proportion of entries into the open arms and the proportion of time in the open arms in SNI rats (*n* = 12 rats/group). **(F,G)** Intraperitoneal injection of 666–15 decreased the eating latency **(F)** and the immobility time **(G)** in SNI rats (*n* = 12 rats/group). **(H)** Intraperitoneal injection of 666–15 suppressed the CREB/BDNF pathway in the spinal cord (*n* = 8 rats/group). **(I)** Intraperitoneal injection of 666–15 decreased the expression of p-CREB, BDNF, and PSD, but not CREB, in the ACC (*n* = 8 rats/group). *P < 0.05, ^**^P < 0.001, ^***^P < 0.0001 for SNI + Control vs. SNI + 666–15.

Clinical applications are mostly through peripheral administration. To further explore whether peripheral modulation of the CREB/BDNF signaling pathway can modulate neuropathic pain and anxiodepression, we chose 666–15 (10 mg/kg), a specific inhibitor of CREB by intraperitoneal injection (Sun B. et al., 2021; Figure 8A). The administration of 666–15 was shown to reverse both mechanical allodynia and thermal hyperalgesia produced by peripheral nerve injury (Figures 8B,C). However, Intraperitoneal injection of 666–15 did not affect PWT and PWL in sham group (Supplementary Figure 3G). Emotional function behavioral tests showed that intraperitoneal injections of 666–15 also reduced anxiety and depression associated with neuropathic pain at 21 days after SNI (Figures 8D–G). At the same time, there was no significant change in the behavioral test of emotional function in sham + 666–15 group (Supplementary Figures 3H–L). Western blotting revealed decreased expression of CREB, p-CREB, BDNF and PSD-95 in the spinal cord of rats in the SNI + 666–15 group (Figure 8H). The expression of p-CREB, BDNF, and PSD-95 was also significantly decreased in the contralateral ACC, but CREB was not altered (Figure 8I), possibly due to the inability of 666–15 to permeate the blood-brain barrier (BBB). At the same time, the injection of 666–15 did not affect the phosphorylation degree of CREB (Supplementary Figure 3E). In summary, 666–15 can inhibit the CREB/BDNF signaling pathway in the spinal cord, block the transmission of pain signals upward at an early stage and then alter the CREB/BDNF signaling pathway in the ACC. This result suggests that early intervention for neuropathic pain, which inhibits the development of pain, may reverse the appearance of comorbidities, including anxiety and depression.

### Lipopolysaccharide Intraperitoneally Activated the cAMP Response Element-Binding Protein/Brain-Derived Neurotrophic Factor Pathway in the Anterior Cingulate Cortex

**FIGURE 9:**
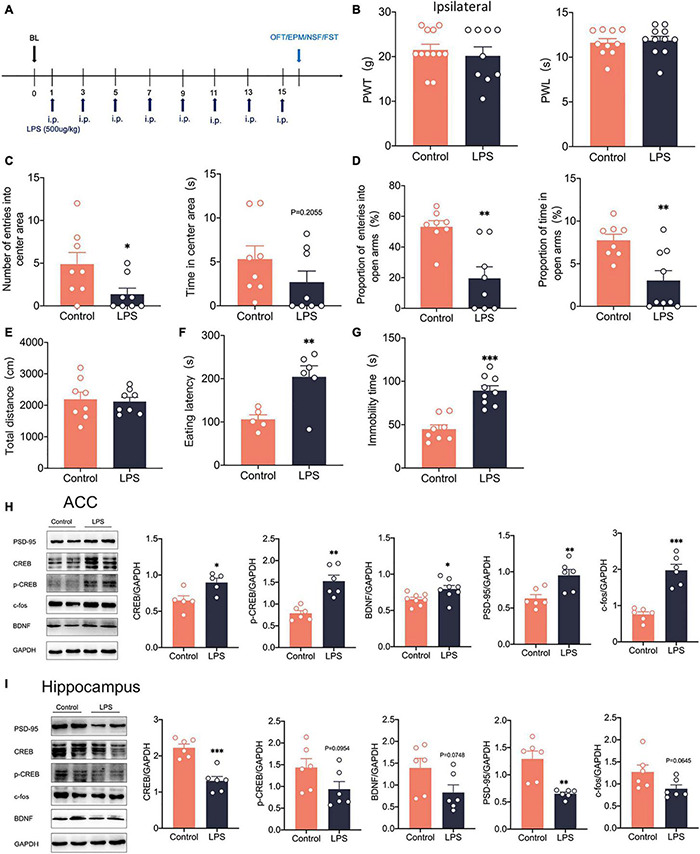
Intraperitoneal administration of LPS induced anxiodepression like behaviors in rats and activated the CREB/BDNF signaling pathway in the ACC. **(A)** Lipopolysaccharide (LPS) was injected intraperitoneally (i.p.) every 2 days for 14 days. **(B)** Intraperitoneal administration of LPS did not affect PWT and PWL in rats (*n* = 9 rats/group). **(C)** Intraperitoneal administration of LPS reduced the number of entries into the center area, but not the time in the center area in rats (*n* = 9 rats/group). **(D)** Intraperitoneal administration of LPS reduced the proportion of entries into the open arms and the proportion of time in the open arms in rats (*n* = 9 rats/group). **(E)** Intraperitoneal administration of LPS did not alter the locomotor activity of the rats (*n* = 9 rats/group). **(F,G)** Intraperitoneal administration of LPS increased the eating latency **(F)** and the immobility time **(G)** in rats (*n* = 9 rats/group). **(H)** Intraperitoneal administration of LPS activated the CREB/BDNF pathway in the ACC (*n* = 6 rats/group). **(I)** Intraperitoneal administration of LPS Suppressed the CREB/BDNF pathway in the hippocampus (*n* = 6 rats/group). *P < 0.05, ^**^P < 0.001,^***^P < 0.0001 for Control vs. LPS.

To examine how anxiety and depression comorbid by neuropathic pain differ from pure anxiety and depression, We constructed the depression model rats by intraperitoneal injection of lipopolysaccharide (LPS, 500 μg/kg) into the rats (Rubab et al., 2021; Figure 9A). After 14 days, we found that PWT and PWL did not change significantly in LPS rats (Figure 9B). However, significant anxiety and depression-like behaviors were observed in LPS rats (Figures 9C–F). LPS injected intraperitoneally also did not affect the locomotor activity of the rats (Figure 9G). Western blotting showed that c-fos expression was elevated in the ACC (Figure 9H), indicating that intraperitoneal injection of LPS activated the ACC. CREB, p-CREB, BDNF and PSD-95 expression were also significantly increased in the ACC of LPS rats (Figure 9H). However, CREB, p-CREB, BDNF and PSD-95 expressions were significantly decreased in the hippocampus of LPS rats compared with the control rats (Figure 9I), which was similar to what we observed in the SNI model. At the same time, the injection of LPS did not affect the phosphorylation degree of CREB (Supplementary Figure 3F). In summary, the depression model was constructed by intraperitoneal injection of LPS, which activated the ACC and the CREB/BDNF pathway in the ACC. However, the CREB/BDNF pathway was suppressed in the hippocampus. We speculate that the inhibition of CREB/BDNF pathway in hippocampus may be a necessary condition for the expression of depression, whether in neuropathic pain or LPS induced depression models.

## Discussion

Neuropathic pain resulting from peripheral nerve injury is often accompanied by cognitive, memory, and emotional dysfunction. Studying the pathogenesis of comorbidities may better help us understand how neuropathic pain is altered. We chose the selective nerve injury (SNI) model to mimic clinical neuropathic pain and found that SNI rats developed pain hypersensitivity and anxiodepression in the late phase of neuropathic pain. In parallel, the spinal cord and ACC are highly activated, and we found that pain hypersensitivity and anxiodepression were alleviated in SNI rats after silencing the ACC by chemicalgenetic techniques. The CREB/BDNF pathway was also activated in the ipsilateral spinal cord and contralateral ACC. The anxiety and depression were alleviated in SNI rats after specific knockdown of CREB by stereotaxic injection of adeno-associated virus (AAV)—delivered shRNA in the ACC. In addition, peripheral administration of CREB inhibitor-666–15 alleviated the transmission of pain signals and then suppressed the occurrence of comorbidity by intervening in the CREB/BDNF pathway in the spinal cord. Therefore, the current study confirms the critical role of the ACC in neuropathic pain and anxiodepression. We next explore the underlying mechanisms of central and peripheral CREB/BDNF involvement in comorbidity by combining previous studies.

The idea that chronic pain, including neuropathic pain, causes cognitive, memory, and emotional functions impairment are well recognized (Zhang et al., 2021). The immobility time of rodents in the FST was significantly increased at 30–35 days after a chronic constriction sciatic nerve injury (CCI) model (Bai et al., 2021; Llorca-Torralba et al., 2021). In summary, depression-like behaviors appear at late stages of neuropathy. In the SNI model, the study finds a significant increase in the eating latency and immobility time of rats at 14 days after SNI. However, the proportion of preference for sugar water does not show significant differences until 35 days (Mecca et al., 2021), suggesting that the time of the appearance of emotional dysfunction may differ in different neuropathic pain models. For behavioral assays, we selected four time points, 7, 14, 21, and 28 days after SNI. In addition, all behavioral tests are separated from each other to reduce mutual effects. At 14 days, SNI rats show anxiety-like behavior in the OFT, EPM, and NSFT, but not depression-like behavior. However, at 21 days after surgery, SNI rats significantly increase in the eating latency in the NSFT and the immobility time in the FST, indicating depression-like behavior. To initially evaluate the probability of neuropathic pain-induced emotional dysfunction, we assessed the SNI rats using the mean of each experimental index value of sham rats as a reference. The results show that 86% of the SNI rats develop anxiety and depression-like behaviors at 21 days. Overall, the SNI model induces anxiety-like behaviors in rats at an earlier period after surgery, whereas the appearance of depression-like behaviors may occur at a later period.

ACC, the anterior cingulate cortex, is an essential cortical region involved in pain signaling and emotion regulation. Peripheral nerve injury, which promotes the upward transmission of nociceptive signals from the spinal dorsal horn to the ACC, leads to activation of structure and function in the ACC (Wang et al., 2020). Descending inhibition of pain signaling is exerted in the projection of ACC -periaqueductal gray (PAG)—rostral ventromedial medulla (RVM) (Oliva et al., 2021). Recently, Chen et al. (2018) showed that ACC could project directly to the spinal dorsal horn and facilitate transmitting pain signals. To validate the anatomical relationship between the spinal cord and ACC, we perform trans-monosynaptic retrograde tracing by CTB. We injected CTB-555 into the ipsilateral dorsal horn along with CTB-488 into the contralateral ACC. Immunofluorescence reveals that the colabeling signal is observed in the ipsilateral dorsal horn and the contralateral ACC, suggesting an anatomical projection between the spinal cord and ACC. At the same time, the spinal cord and ACC are highly activated, and the synapse establishment is increased after peripheral nerve injury. To further validate the role of the ACC in neuropathic pain comorbid with emotional dysfunction, we applied chemogenetic approaches to extensively silence neurons in the ACC and showed that intraperitoneal administration of CNO significantly alleviates pain allodynia, while also reversing depression-like behaviors as well as anxiety in SNI rats, indicating that the ACC is activated in SNI rats and involved in the transmitting pain and emotional signals, whereas silencing the ACC can reduce neuropathic pain and emotional dysfunction.

The CREB/BDNF pathway, which regulates neuronal growth, differentiation and synapse establishment, has been implicated in several neurological disorders (Wang I. et al., 2021; Xie et al., 2021). Since our previous study found that elevated CREB in DRG is involved in paclitaxel-induced chemotherapeutic pain (Yang Y. et al., 2021) we want to explore the effects of peripheral nerve injury on CREB/BDNF signaling in the spinal cord and ACC. The results show that the expression of CREB and BDNF was enhanced in the ipsilateral spinal cord of SNI rats. P-CREB, which has also been used as a marker molecule for NMDA receptor-dependent long-term potentiation (LTP) (Carrano et al., 2019), and CaMKIIα, as an essential protein kinase for CREB phosphorylation and excitatory neuronal synaptic marker (Moro et al., 2020), are also significantly increased after SNI in the ipsilateral spinal cord. Taken together, the above results indicate that after peripheral nerve injury, the CREB/BDNF pathway is activated in the spinal cord, which may contribute to the convergence and transmission of pain signals to the spinal cord by altering synapse establishment and function, similar to previous studies (Fujimura et al., 2021). At the same time, the expression of CREB, p-CREB, and CaMKIIα in the ACC increase significantly after SNI, following a similar trend as in the spinal cord.

Interestingly, our results showed that BDNF expression is not significantly changed at 28 days after SNI, but the BDNF mRNA is still increased. At the same time, the CREB/BDNF pathway is significantly decreased in the SNI rats with comorbid anxiety and depression. In summary, we speculate that in neuropathic pain condition, increased BDNF expression promote synapse establishment and remodeling in the ACC. However, when combined with anxiety and depression, the secreted BDNF in the ACC, including the hippocampus to maintain the homeostasis of BDNF in the brain. In summary, activation of the CREB/BDNF pathway in the ACC drives pain signals to converge into the ACC, thereby mediating the transmission of the pain and emotional components of nociceptive signals to the spinal cord and other brain regions.

To investigate the effects of specifically inhibiting the CREB/BDNF pathway in the ACC on neuropathic pain and emotional dysfunction, we specifically knocked down CREB in the ACC. We found that knockdown of CREB in the ACC suppresses the expression of BDNF and increases the PWT and PWL in SNI rats. It also alleviates anxiety and depression-like behaviors in SNI rats. Surprisingly, CREB/BDNF knockdown in the ACC significantly increases CREB and BDNF in the spinal cord, and the increased BDNF may exert its neurotrophic effects on the repair of injured peripheral nerves (Ferrini et al., 2020). The intervention on the CREB/BDNF pathway in the ACC will not only relieve neuropathic pain but also alleviate the emotional dysfunction that accompanies the neuropathic pain, indicating that emotional dysfunction, induced by neuropathic pain (Thibault et al., 2014), may share common pathways and that the CREB/BDNF pathway may be one of the expected targets.

To examine how anxiety and depression comorbid by neuropathic pain differ from pure anxiety and depression, we injected LPS intraperitoneally into rats to construct a depression model. In the LPS model, pain sensitivity is not significantly altered, but it induces significant anxiety and depression-like behaviors (Li et al., 2018). C-fos expression is significantly elevated in the ACC, indicating that LPS injected intraperitoneally activates the ACC. Meanwhile, the CREB/BDNF pathway is also activated in ACC. Our results showed that the CREB/BDNF pathway was inhibited in the hippocampus of LPS rats. This result is consistent with what we observed in the SNI model, which shows that there are commonalities between anxiodepression induced by neuropathic pain and alone anxiodepression. The potential mechanism of LPS in constructing depression model may be to promote the cascade inflammatory response in rats, similar to sepsis. CREB/BDNF pathway is also involved in the production and communication of inflammatory factors (Shen et al., 2022). Therefore, CREB/BDNF pathway may also be due to the regulation of inflammatory factors involved in peripheral nerve injury and emotional dysfunction. However, there may be differences in the mechanisms that occur between different models of depression, including the chronic unpredictable mild stress model (CUMS), and therefore the results obtained may also differ. However, in general, CREB/BDNF in rat hippocampus decreased in SNI model, LPS model and CUMS model. We speculate that the inhibition of CREB/BDNF pathway in hippocampus may be a necessary condition for the expression of depression.

Whether pain-induced anxiety and depression will exist independent of pain is also a question to be considered. We take CREB/BDNF as a target to explore whether inhibiting the development of pain will avoid the occurrence of depression-like behaviors as well as anxiety. Administration of 666–15, a CREB specific inhibitor but unable to permeate the blood-brain barrier (Yang C. et al., 2021), significantly attenuates mechanical allodynia and thermal hyperalgesia, and reverses anxiety and depression-like behaviors in SNI rats. Meanwhile, the CREB/BDNF pathway and excitatory synapse establishment decreased in the spinal cord. P-CREB, BDNF, and PSD-95 decreased in the ACC, while CREB did not change, so that 666–15 suppresses upward transmission of pain signals in the spinal cord, blocks activation of the CREB/BDNF signaling pathway in the ACC, and prevents the progress of mood disorders (Sun B. et al., 2021). There are still several limitations to our experiments, we only employ synaptic markers to evaluate synapse construction and fail to employ electrophysiological techniques to evaluate functional changes at synapses. Whether activation of the CREB/BDNF pathway in the ACC can induce pain allodynia in rats requires further investigation and exploration. Also, what kind of neurons in the ACC is activated by peripheral nerve injury and the circuit regulation of inhibitory and excitatory neurons within the ACC will be the focus on future studies.

In summary, we demonstrate that neuropathic pain in the late phase is comorbid with anxiety and depression. The spinal cord and ACC are highly activated in comorbidities, and silencing the ACC alleviates neuropathic pain and anxiety and depression-like behaviors. Meanwhile, CREB/BDNF is also activated in the spinal cord and ACC, which promotes synapse establishment and remodeling, thereby mediating the transmission of the nociceptive and emotional components of pain signaling, and representing one of the critical targets to study neuropathic pain and comorbidities.

## Materials and Methods

### Animals

Adult male, Sprague Dawley rats, weighing 200–250 g and aged 6–8 weeks (Sbeff bioscience Co., Ltd., Beijing, China) were kept on a 12 h light/dark cycle with full access to water and food. Attempts have been made to keep the number of rats utilized to a minimal necessary for statistical accuracy while minimizing their suffering. Rats were randomized to various experimental groups at random. The rats were sedated with an intraperitoneal injection of pentobarbital sodium (40 mg/kg) when surgical operations were done. The techniques were compliant with the National Institutes of Health’s Guide for the Care and Use of Laboratory Animals and were approved by Zhengzhou University’s Ethical Committee for Animal Research (No. 2021060).

### Animal Models

#### Spared Nerve Injury Surgery

To simulate clinical neuropathic pain, we used a previously established sparing nerve injury (SNI) model (Zhao et al., 2017). I In summary, rats were anesthetized with pentobarbital sodium (40 mg/kg) intraperitoneally. Under aseptic surgical settings, the left sciatic nerve branches were separated through blunt dissection of the femoral biceps muscle. Both the peroneal and tibial nerves were ligated firmly and distal to the ligation, leaving the sural nerve intact. Following surgery, the surrounding muscle and skin were sutured and sterilized. All previous operations were performed on sham-operated animals lacking nerve ligation or transection. Seven, 14, 21, and 28 days following surgery, behavioral tests were done.

#### Lipopolysaccharide Induced Depression Model

For the depression model, the rats received an intraperitoneal injection of lipopolysaccharide (LPS) (500 μg/kg, No: L2880, Millipore Sigma Co., Ltd., United States) every 2 days for 14 days as described previously (Rubab et al., 2021). A control group was exposed to DMSO at the same dose. Behavioral tests were conducted at 14 days after LPS injection.

#### Behavioral Analyses

These behavioral analyses were performed on awake, unrestrained, age-matched rats. Behavioral testing was conducted on rats by an investigator who was unaware of the groups’ identities. Mechanical allodynia was examined using the paw withdrawal threshold (PWT) in response to the manual application of graded von Frey hairs (1.4–26 g) to the plantar surface. The thermal hypersensitivity of the paw withdrawal latency (PWL) to a radiant heat source applied to the plantar surface was determined. Anxiodepressive-like behaviors were examined increased forced swimming experiment (FST), plus-maze test (EPM), novelty suppressed feeding test (NFST), and with open field test (OFT).

#### Paw Withdrawal Threshold

Three days before to the experiments, all rats were put in a Plexiglas cage with a metal net bottom for 30 min every day. After acclimatization to the surroundings, the hind paw was stimulated using a succession of von Frey hairs with logarithmically increasing stiffness (1.4, 2, 4, 6, 8, 10, 15, and 26 g) (Aesthesio, United States). A force sufficient to bend the filament perpendicular to the plantar surface was applied for 5 s. If the hind paw was promptly and totally lifted off the platform, a favorable outcome was documented. We determined the withdrawal threshold using the up-down method (Chaplan et al., 1994).

#### Paw Withdrawal Latency

Individual test compartments (7,370, Ugo Basile, Comeria, Italy) were put on a temperature-controlled glass platform maintained at 30°C, and the lateral plantar surface of the hind paw was stimulated using a radiant heat source delivered via an aperture. Thermal paw withdrawal delay was defined as the time interval between stimulus commencement and paw withdrawal. Five times at 5-min intervals, each hind paw was evaluated, and the pullout delay data were averaged. To minimize tissue damage caused by extended heat stimulation, the cutoff latency was chosen to 15 s (Hargreaves et al., 1988).

#### Open Field Test

These tests were carried out in the dark phase, the light intensity was maintained at the same level throughout the experiment, and the rats were acclimatized to the testing room for 30 min prior to the commencement of the testing to guarantee steady activity throughout the trial. A 100 × 100 × 40 cm3 box served as the open field test chamber. At the start of the experiment, the rats were put in the center zone, and a 5-min video recording of their behavior was done. The test chamber was completely cleaned with 70% ethanol in between the two operations to eliminate any lingering scents. The frequency of entrances into the center region, the amount of time spent in the central area, and the overall distance were all recorded. The parameters were analyzed using the Smart v.3.0 software (Panlab Harvard instruments, Newbury Park, CA, United States).

#### Elevated Plus Maze Test

These assays were carried out in the dark phase, the light intensity was controlled under the same conditions, and the rats were acclimatized to the testing room for 30 min prior to the experiment to ensure stable activity during the test. The test was conducted in a maze consisting of 2 open arms (110 × 10 cm2) and 2 enclosed arms (110 × 10 × 30 cm3) from the central platform (10 × 10 cm2) extending at 90°. All rats were acclimated for 5 min in the open field before the start of the experiment. Initially, the experiment, the rats were placed on a central platform facing the open arm. The activities of rats were recorded by video for 5 min. Between the two processes, the maze was thoroughly cleaned with 70% ethanol to remove residual odors. The measured parameters include the number and time of entering the open arm area and the total movement distance. Smart v.3.0 software (Panlab Harvard instruments, Newbury Park, CA, United States) was used to analyze these parameters. The proportion of entries into the open arms = the number of entries into the open arms/ (the total number of entries into the open arms and the enclosed arms); The proportion of time in the open arms = the time in the open arms/(the total time in the open arms and the enclosed arms).

#### Novelty Suppressed Feeding Test

These assays were carried out in the dark phase, the light intensity was controlled under the same conditions, and the rats were acclimatized to the testing room for thirty minutes prior to the experiment to ensure stable activity during the test. The new environment feeding inhibition experiment chamber was a 100 × 100 × 40 cm3 box. A small piece of white paper was placed in the center of the box bottom, and small pieces of food were put on the white paper. Animals were fasted for 24 h before the experiment and the rats were placed into a corner area at the beginning of the test. The rats began to bite and the timing ended. Between the two processes, the box was thoroughly cleaned with 70% ethanol to remove residual odors. The time that rats spent from placement to initiation of eating were recorded. If the rat did not eat after 10 min, the data was deleted.

#### Forced Swimming Experiment

The rats were placed into a cylindrical gum cylinder with a diameter of 20 cm and height of 60 cm, with a water depth of 30–35 cm and a water temperature of 23–25^°^C. The total forced swim time was 6 min, and the first minute was set as the acclimation time. The immobility time was recorded within 5 min after that. The water was changed between each animal to prevent odors from affecting later rats. The criteria for determining immobility were as follows: Rats stopped struggling in the water and exhibited floating condition, or only fine limb movements to keep the head floating on the water. Two persons with unclear grouping performed experimental data recording.

### Western Blotting

Briefly, the rats were decapitated at a predetermined timepoint. The contralateral ACC and ipsilateral spinal cord were taken and placed in liquid nitrogen for a short period of time. After homogenizing the tissues in ice-cold RIPA lysis buffer (#CW2333, CWBIO, Beijing, China) supplemented with 0.1 mM phenylmethylsulphonyl fluoride (PMSF)-protease inhibitors (#CW2200S, CWBIO, Beijing, China) and phosphatase inhibitors (#CW2383S, CWBIO, Beijing, China), the samples were analyzed. Centrifuge the crude homogenate at 4°C for 15 min at 3,000 r/min, and collect the supernatants. After determining the protein concentrations, the samples were heated to 99°C for 5 min, and 20–40 g protein was loaded onto 10% SDS-polyacrylamide gels. Electrophoretically, the proteins were transferred to PVDF membranes. After blocking the membranes with 3% non-fat milk for 1 h, they were incubated overnight at 4°C with the primary antibody. The proteins were identified using anti-mouse or anti-rabbit secondary antibodies coupled to horseradish peroxidase, and the bands were seen using enhanced chemiluminescence (ECL, Thermo Fisher Scientific United States) and detected using a ChemiDoc™ machine (Bio-Rad, United States). The relative levels of the target protein were measured by conducting a densitometry analysis through the Image J program, normalized to the levels of -actin (National Institutes of Health, United States). Table 1 lists the antibodies that were employed.

**TABLE 1:** The primary antibodies for western blotting.

Antibody	Company	Dilution
c-fos	Cat No. 0469R, Bioss, Inc., Beijing, China	1:1,000
CREB	Cat no. 9197S, Cell Signaling Technology, Inc., United States	1:1,000
p-CREB	Cat no. 9198S, Cell Signaling Technology, Inc., United States	1:1,000
PSD-95	Cat no. 3450S, Cell Signaling Technology, Inc., United States	1:1,000
CaMKII α	Cat no. 5049S, Cell Signaling Technology, Inc., United States	1:1,000
NLGN2	Cat no. ab177107, Abcam, United Kingdom	1:2,000
BDNF	Cat no. ab108319, Abcam, United Kingdom	1:1,000
GAPDH	Cat no. 60004-1-Ig, Proteintech Group, Inc., Wuhan, China	1:5,000
Goat anti-rabbit IgG	Cat no. SA00001-2, Proteintech Group, Inc., Wuhan, China	1:5,000
Goat anti-mouse IgG	Cat no. SA00001-1, Proteintech Group, Inc., Wuhan, China	1:5,000

### Immunofluorescence Labeling

Following defined survival durations, the rats were terminally anesthetized and perfused with normal saline via the ascending aorta, then immersed in 4% paraformaldehyde in 0.1 M phosphate buffer. Following perfusion, the spinal cord and ACC were removed and postfixed for 12 h in the same fixative, subsequently replaced with 20, 30, and 40% sucrose phosphate buffered saline throughout one night. Transverse spinal cord (20 m) and ACC (20 m) slices were produced for immunofluorescence staining using a cryostat (Leica, CM1950). After washing with PBS, the sections were blocked for 1 h at 37°C with 5% goat serum in 0.3 percent Triton X-100 and incubated overnight at 4°C with the primary antibody. The sections were treated for 2 h at 37°C with a combination of goat anti-mouse Cy3-conjugated secondary antibody (1:200, Jackson ImmunoResearch, Amish, PA) and goat anti-rabbit FITC-conjugated secondary antibody (1:400, Jackson ImmunoResearch). ECLIPSE Si was used to visualize the staining of the slices (Nikon, Japan). The primary antibodies listed in Table 2 were utilized.

**TABLE 2:** The primary antibodies for Immunofluorescence.

Antibody	Company	Dilution
GFAP	Cat no. 3670S, Cell Signaling Technology, Inc., United States	1:300
CREB	Cat no. 9197S, Cell Signaling Technology, Inc., United States	1:500
p-CREB	Cat no. 9198S, Cell Signaling Technology, Inc., United States	1:200
PSD-95	Cat no. 3450S, Cell Signaling Technology, Inc., United States	1:200
Fos B	Cat no. ab11959, Abcam, United Kingdom	1:200
BDNF	Cat no. ab108319, Abcam, United Kingdom	1:200
Iba-1	Cat no.GT10312, Thermo Fisher Scientific, United States	1:200
Neun	Cat no. 66836-1-Ig, Proteintech Group, Inc., Wuhan, China	1:200
Goat anti-mouse Cy3	Cat no. 115-165-003, Jackson ImmunoResearch, United States	1:200
Goat anti-rabbit FITC	Cat no. 115-095-003, Jackson ImmunoResearch, United States	1:200

### Quantitative RT-PCR

After decapitating the animals at a predetermined time point, we extracted the ipsilateral spinal cord and contralateral ACC tissues and shock-froze them on dry ice. Total RNA was isolated from spinal cord and ACC tissue according to the manufacturer’s procedure by TRIzol Reagent (Cat. No. DP419, TIANGEN Biochemical Technology Co., Ltd., Beijing, China). PrimeScript RT Master Mix (Cat. No. RR047A, TaKaRa, Dalian, China) was employed to reverse transcribe RNA extracts at 37°C for 15 min and 85°C for 5 s. The targets were then amplified in triplicate using TB Premix Ex Taq (Catalog No. RR420A, TaKaRa, Dalian, China) on an Applied Biosystems StepOnePlus Real-Time PCR System (Foster City, CA, United States), normalized to GAPDH, and quantified using the comparative cycle threshold technique (2CT). Table 3 contains primers sequences.

**TABLE 3:** The primers for Quantitative RT-PCR.

Gene	Forward	Reverse
CREB	GGAGCAGACAACCAGCAGAGTG	GGCATGGATACCTGGGCTAATGTG
BDNF	ACGGCAAGTTCAACGGCACAG	CGACATACTCAGCACCAGCATCAC
GAPDH	TGGAACTCGCAATGCCGAACTAC	TCCTTATGAACCGCCAGCCAATTC

### Drug Infusion

CREB inhibitor 666–15 (Cat No.HY-101120, MCE, New Jersey, United States) was administered by intraperitoneal injection at the above time point before operation and the day before behavioral test according to the dose of 10 mg/kg in the literature (Yang C. et al., 2021). It should be noted that the drug can be administered only when there is no blood. After administration, the rats were returned to the animal room after 30 min of observation.

### Stereotaxic Surgery

Isoflurane was used to anesthetize rats, and their heads were secured in a stereotactic frame (RWD Life Science Inc., Shenzhen, China). To avoid corneal dryness, erythromycin was given to their eyes. All skull measurements were taken in relation to Bregma, and the virus was injected into the ACC at a rate of 50 nl/min using a 5–l microsyringe (Gaoge, Shanghai, China) equipped with a microelectrode. Following viral injection, the microelectrode was left *in situ* for 10 min to allow for virus diffusion. The anterior posterior (AP) injection site was 1.35 mm, the medial lateral (ML) injection site was 0.55 mm, and the dorsal ventral (DV) injection site was 2.25 mm. For the CREB gene knockout experiment, BrainVTA Technology (Wuhan, China) formed and implanted a short hairpin RNA (shRNA) targeting Creb1 into an adeno-associated virus (AAV) vector expressing enhanced green fluorescent protein (EGFP); this shRNA specifically targets rat neurons and empowers significant down-regulation of Creb1 expression. 300 nl of AAV2/9-U6-shRNACreb1-CMV EGFP orAAV2/9-U6-Scramble-CMV-EGFP was injected into the contralateral ACC. For retrograde tracing experiments, 250 nl CTB-555 (BrainVTA Biotechnology Co., Ltd., Wuhan, China) was injected into the ipsilateral spinal cord and 250 nl CTB-488 into the contralateral ACC. The dates were excluded from experiments when viral injections were inaccurate.

### Chemical Heredity

Two weeks before SNI, 200 nl of the chemogenetic virus rAAV-hSyn-hM4D (Gi)-EGFP-WPRE-pA (Cat No.PT-0153, BrainVTA Biotechnology Co., Ltd., Wuhan, China) was injected into the contralateral ACC using the brain stereotaxic microinjection technique as described above. Clozapine N-oxide (CNO, BrainVTA Biotechnology Co., Ltd., Wuhan, China) 3.3 mg/kg was injected intraperitoneally and behavioral tests were performed 45 min after injection (Huijgens et al., 2021).

## Conclusion

In conclusion, peripheral nerve injury abnormally activates the spinal cord and ACC, which allows the transmission of nociceptive signals from the periphery to the center and causes central sensitization, and induces neuropathic pain and comorbid anxiodepression-like behaviors. Non-specific silencing of ACC by a chemical genetic method can alleviate neuropathic pain and anxiodepression-like behaviors in SNI rats, indicating that abnormal activation of ACC is involved in it. The CREB/BDNF pathway was also activated in the spinal cord and ACC in SNI rats. Inhibiting the CREB/BDNF pathway centrally and peripherally, respectively, can inhibit the abnormal activation of spinal cord and ACC, which alleviates neuropathic pain and anxiodepression-like behaviors in SNI rats, indicating that the CREB/BDNF pathway may be a potential mechanism of ACC activation.

## Data Availability Statement

The original contributions presented in the study are included in the article/Supplementary Material, further inquiries can be directed to the corresponding author/s.

## Ethics Statement

The animal study was reviewed and approved by Ethics Committee of the Second Affiliated Hospital of Zhengzhou University.

## Author Contributions

JW and YX: experimental design, experimental conduct, data collection, data processing, and original article writing. ZY: experimental conduct, data collection, and data processing. YZ, WW, JY, and YW: experimental conduct, data collection. QB and ZL: experimental design, experimental conduct, article revision, and article review. All authors read and approved the final manuscript.

## Conflict of Interest

The authors declare that the research was conducted in the absence of any commercial or financial relationships that could be construed as a potential conflict of interest.

## Publisher’s Note

All claims expressed in this article are solely those of the authors and do not necessarily represent those of their affiliated organizations, or those of the publisher, the editors and the reviewers. Any product that may be evaluated in this article, or claim that may be made by its manufacturer, is not guaranteed or endorsed by the publisher.
