# Supplementary material for: The cAMP Response Element- Binding Protein/Brain-Derived Neurotrophic Factor Pathway in Anterior Cingulate Cortex Regulates Neuropathic Pain and Anxiodepression Like Behaviors in Rats
Source: Front Mol Neurosci. 2022 Mar 24;15:831151. doi: 10.3389/fnmol.2022.831151 (PMC8987281; doi:10.3389/fnmol.2022.831151)
Supplement: Supplementary file 3 [file Image_1.pdf]

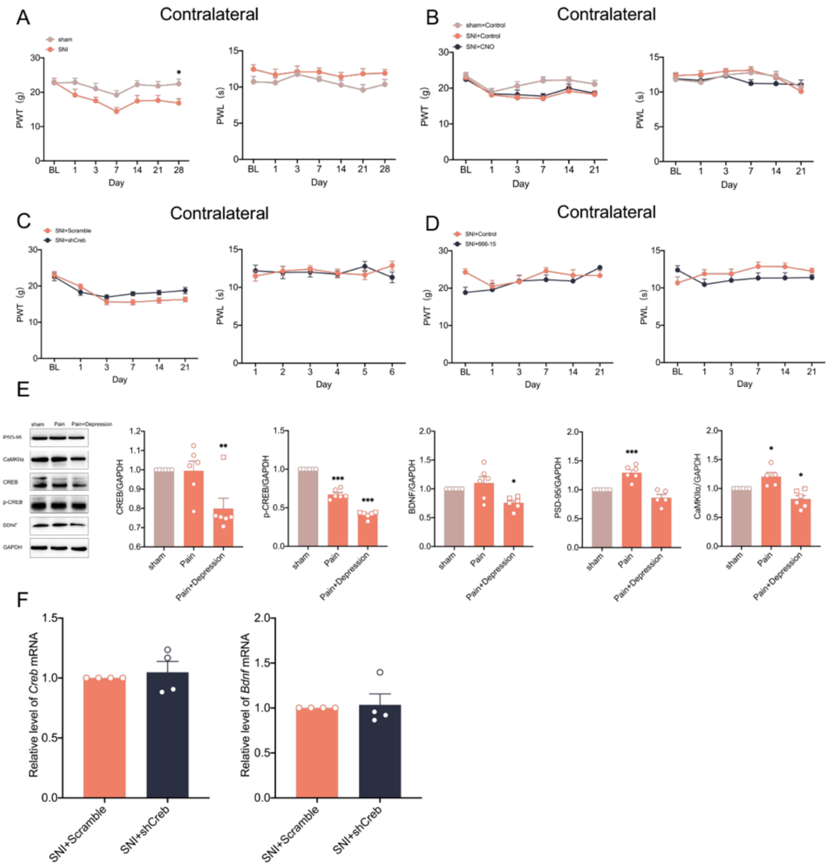

Supplemental Figure 1:

(A) SNI did not affect PWT and PWL of the contralateral paw in the contralateral paws (n=8 rats/group, $^{*}P < 0.05$ for sham vs. SNI).

(B) Silencing ACC did not significantly affect the PWT and PWL of the contralateral paws in SNI rats (n=10 rats/group).

(C) Knockdown of CREB in the ACC did not significantly affect the PWT and PWL of the contralateral paws in SNI rats (n=12 rats/group).

(D) Intraperitoneal injection of 666-15 did not significantly affect the PWT and PWL of the contralateral paws in SNI rats (n=12 rats/group).

(E) CREB / BDNF signaling pathway was suppressed in the hippocampus of peripheral nerve injury induced depressed rats (n=6 rats/group, $^{*}P < 0.05$, $^{**}P < 0.001$, $^{***}P < 0.0001$ for Sham vs. Pain, Sham vs. Pain+Depression).

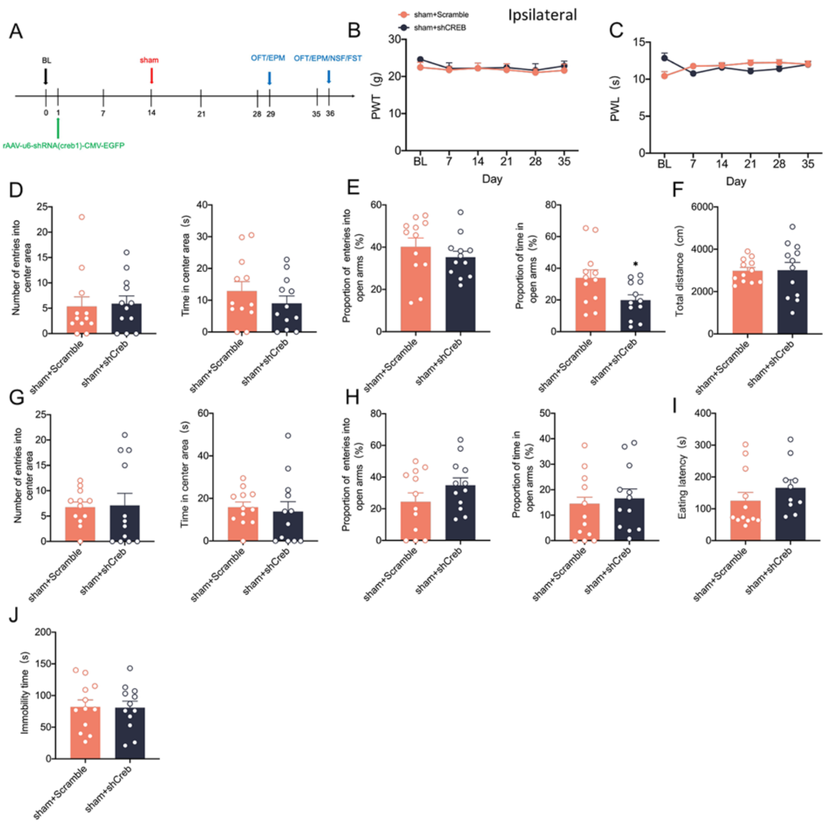

Supplemental Figure 2: Knockdown of CREB in the ACC did not affect pain hypersensitivity and anxiodepression like behaviors in sham rats.

(A) Injection of rAAV-u6-shRNA Creb1-CMV-EGFP into the contralateral ACC of sham rats.

(B and C) Knockdown of CREB in the ACC did not affect PWT and PWL in the sham rats (n=12 rats/group).

(D) Knockdown of CREB in the ACC did not affect the number of entries into the center area and time in the center area in sham rats at 14 d after surgery (n=12 rats/group).

(E) Knockdown of CREB in the ACC decreased the proportion of time in the open arms, but not the proportion of entries into the open arms in sham rats at 14 d after surgery (n=12 rats/group, $^{*}P < 0.05$ for sham+Scramble vs. sham+shCreb).

(F) Knockdown of CREB in the ACC did not affect the locomotor activity of the sham rats (n=12 rats/group).

(G) Knockdown of CREB in the ACC did not affect the number of entries into the center area and the time in the center area in sham rats at 21 d after surgery (n=12 rats/group).

(H) Knockdown of CREB in the ACC did not affect the proportion of entries into the open arms and the proportion of time in the open arms in sham rats at 21 d after surgery (n=12 rats/group).

(I and J) Knockdown of CREB in the ACC did not affect the eating latency (I) and the immobility time (J) in sham rats at 21 d after surgery (n=12 rats/group).

A

c-fos/GAPDH		PSD-95/GAPDH		NLGN2/GAPDH	
sham	SNI	sham	SNI	sham	SNI
0.612247	0.904735	0.713604	1.112156	1.595763	1.410704
0.469224	0.990292	0.877996	1.510103	2.494015	1.380321
0.843712	0.790388	0.825851	1.23001	2.921199	1.267348
0.758296	1.510839	0.657759	1.017039	1.772771	2.512388
0.878781	1.239755	0.682827	1.550841	1.770101	2.061207
0.776954	1.182388	0.821168	1.339536	1.86056	1.526908
0.770206	1.371547	0.852809	1.455254	1.747938	1.480893
	1.54802		1.431321		1.724752

B

c-fos/GAPDH		PSD-95/GAPDH		NLGN2/GAPDH	
sham	SNI	sham	SNI	sham	SNI
0.924216	1.250662	0.603039	1.063264	0.668855	1.250301
0.676402	1.231604	0.630205	1.032805	0.926659	1.392599
0.546745	1.224276	0.768654	1.001858	1.155002	1.155177
0.875108	1.110147	0.613813	1.059875	1.055247	1.551712
0.713068	1.344468	0.744819	1.174235	1.315823	2.434014
0.733746	1.639967	0.71036	1.180327	1.252488	1.889882
0.817274	1.25265	0.708625	1.00284	1.136467	1.735897
0.726279	1.692304	0.819313	1.350353	1.144917	2.247153

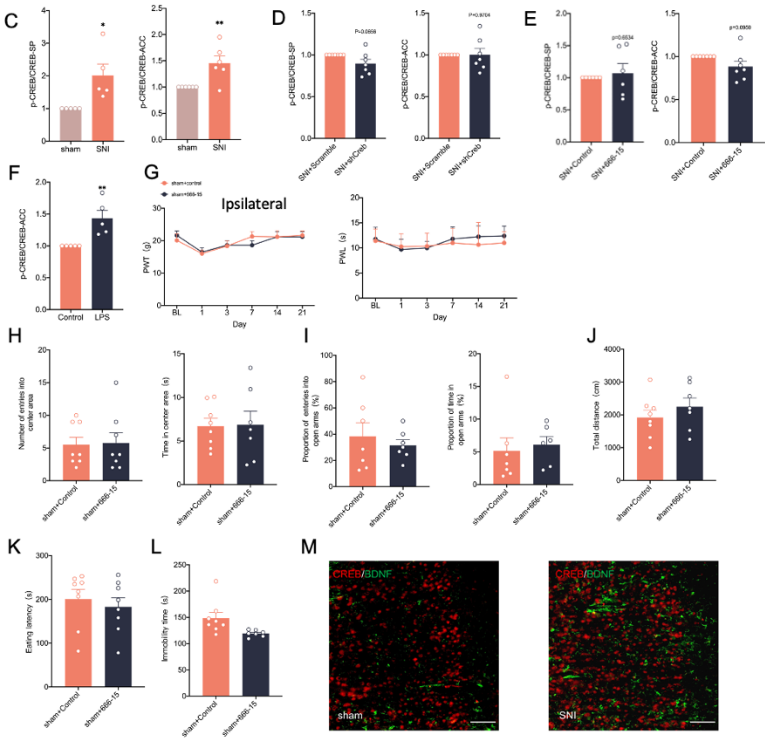

Supplemental Figure 3:

(A and B) The statistical data of Figure 2C (A) and 2D (B).

(C) Changes of CREB phosphorylation in spinal cord and ACC of SNI rats (n=8 rats/group) *P < 0.05, **P < 0.001, ***P < 0.0001 for sham vs. SNI.

(D) Changes of CREB phosphorylation in spinal cord and ACC in SNI + shCreb group (n=8 rats/group).

(E) Changes of CREB phosphorylation in spinal cord and ACC in SNI + 666-15 group (n=8 rats/group).

(F) Changes of CREB phosphorylation in spinal cord and ACC in LPS group (n=8 rats/group). **P < 0.001 for Control vs. LPS.

(G) Intraperitoneal injection of 666-15 did not affect PWT and PWL in sham group (n=8 rats/group).

(H) Intraperitoneal injection of 666-15 did not affect the number of entries into the center area and time in the center area in sham group (n=8 rats/group).

(I) Intraperitoneal injection of 666-15 did not affect the proportion of entries into the open arms and the proportion of time in the open arms in sham group (n=8 rats/group).

(J) Intraperitoneal injection of 666-15 did not affect the total distance in sham group (n=8 rats/group).

(K and L) Intraperitoneal injection of 666-15 did not affect the eating latency (K) and the immobility time (L) (n=8 rats/group).

(M) Immunofluorescence showed that the co labeling of CREB and BDNF in ACC was less in sham group and SNI group. (Scar bar = 50um).
